# Molecular Characterization and Endophytic Colonization of a Native *Beauveria bassiana* Isolate in Maize: Effects on Plant Growth and *Spodoptera frugiperda* Herbivory

**DOI:** 10.3390/biology15131046

**Published:** 2026-07-01

**Authors:** Dulce Betzabeth Rivera-Nuñez, Samuel Pineda-Guillermo, Ana Mabel Martínez-Castillo, Yordanys Ramos

**Affiliations:** Instituto de Investigaciones Agropecuarias y Forestales, Universidad Michoacana de San Nicolás de Hidalgo, Km 9.5, Carretera Morelia-Zinapécuaro, Tarímbaro 58880, Michoacán, Mexico; 1208249j@umich.mx (D.B.R.-N.); samuel.pineda@umich.mx (S.P.-G.)

**Keywords:** biological control, endophyte–plant interaction, entomopathogenic fungi, fall armyworm, crop protection

## Abstract

*Beauveria bassiana* (Balsamo-Crivelli) Vuillemin can colonize plant tissues as an endophyte, promoting plant growth and defense against pathogens and insect herbivory. Understanding the endophytic behavior of native isolates is important for developing pest management strategies in maize, a crop affected by *Spodoptera frugiperda* (J. E. Smith). This study aimed to genetically characterize a Mexican *B. bassiana* isolate (Bb-IIAF1-24) collected in Los Reyes, Michoacán, using the *β-tubulin* gene. The phylogenetic relationships showed that this isolate formed a well-supported clade with other *B. bassiana* isolates from different countries; however, a low divergence among them was observed. In a second part of this study, the influence of foliar and soil inoculation (1 × 10^8^ conidia mL^−1^) of Bb-IIAF1-24 isolate on endophytic colonization in maize plants, as well as the effects on plant growth and herbivory by *S. frugiperda* were evaluated. This fungus was detected in roots, stems, and leaves, but no significant differences were found between root and stem colonization or between foliar and soil inoculation methods. *Beauveria bassiana* treatment resulted in increased stem diameter in plants when applied to soil compared to foliar application and the control. In contrast, plants subjected to foliar application were significantly taller than those receiving soil application or the control plants. Plants from both application methods experienced lower leaf damage compared to the control. These findings demonstrate the potential of the Bb-IIAF1-24 isolate as an endophytic fungus to promote maize growth and reduce herbivory by *S. frugiperda*.

## Simple Summary

*Beauveria bassiana* is a beneficial fungus widely known for its ability to infect and kill insect pests. In addition to this role, it can live inside plant tissues as an endophyte, helping plants grow and increasing their resistance to pests. In this study, we characterized a Mexican isolate of *B. bassiana* (Bb-IIAF1-24) using molecular techniques and evaluated its ability to colonize maize plants. Phylogenetic analyses confirmed that the isolate belongs to *B. bassiana* and is closely related to isolates from other regions of the world. We then inoculated maize plants through either foliar spraying or soil application and assessed fungal colonization, plant growth, and damage caused by the fall armyworm, *Spodoptera frugiperda*, a major maize pest. The fungus successfully colonized roots, stems, and leaves, with the highest colonization levels observed in leaves. Soil application increased stem diameter, while foliar application promoted greater plant height. Both inoculation methods reduced leaf damage caused by the insect compared with untreated plants. These findings demonstrate that the Mexican isolate Bb-IIAF1-24 can establish as an endophyte in maize and provide benefits related to plant growth and protection against insect herbivory, highlighting its potential for sustainable crop management.

## 1. Introduction

Maize (*Zea mays* L.) is one of the most important staple crops worldwide, playing a key role in food security and agricultural systems [1,2]. However, maize production is severely affected by insect pests, among which the fall armyworm, *Spodoptera frugiperda* (J. E. Smith) (Lepidoptera: Noctuidae), is one of the most destructive species [3]. Although this insect is native to the Americas, it is currently distributed cross many regions of the world [4]. The fall armyworm can cause between 45 and 100% of yield losses due to its high feeding capacity and adaptability, particularly during early plant developmental stages [5,6]. At present, management strategies for *S. frugiperda* rely heavily on chemical insecticides, which can cause environmental issues, non-target effects, and the development of resistance [7,8,9]. In this context, entomopathogenic fungi, such as *Beauveria bassiana* (Balsamo-Crivelli) Vuillemin, have gained attention as environmentally friendly alternatives for pest management [10]. Beyond their role as biological control agents, entomopathogenic fungi can establish as endophytes within plant tissues without causing disease, providing additional benefits to the host plant [11,12]. Endophytic colonization by *B. bassiana* has been associated with enhanced plant growth, increased tolerance to abiotic stress, and reduced herbivory by insect pests [13,14,15]. These effects are thought to arise from direct interactions with herbivores or through indirect mechanisms involving changes in plant physiology or induced defenses [16,17,18]. However, several limitations have restricted the widespread use of endophytic entomopathogenic fungi in integrated pest management (IPM) programs. For example, successful colonization of plant tissues by these fungi may require time for establishment, potentially delaying their immediate effectiveness and influencing their persistence as endophytes [19,20,21]. In addition, the efficacy of these pathogens depends on several factors, including the fungal isolate, plant species, and inoculation method [22]. Soil and foliar applications are commonly employed to introduce these microorganisms into plants; however, they may differ in their efficiency and the spatial distribution of fungal colonization within plant tissues [23]. Understanding how these application methods influence fungal establishment and plant responses is essential for optimizing their application under agricultural conditions.

Additionally, the use of native entomopathogenic fungi isolates may offer advantages in terms of adaptation to local environmental conditions and compatibility with the host plant [24]. Therefore, the accurate identification of native isolates is crucial to ensure their taxonomic assignment and support their potential use in biological control programs. We hypothesized that the native Bb-IIAF1-24 isolate would successfully establish as an endophyte in maize tissues, and that its colonization pattern would vary according the inoculation method. Finally, we expected that endophytic colonization of this isolate can promote plant growth and reduce herbivory by *S. frugiperda*.

The present study aimed to confirm the identity of a Mexican *B. bassiana* isolate using the *β-tubulin* gene. The influence of foliar and soil inoculation methods on endophytic colonization in maize plants, and their effects on plant growth and herbivory by *S. frugiperda*, were also evaluated.

## 2. Materials and Methods

### 2.1. Insect Rearing

*Spodoptera frugiperda* larvae were obtained from a laboratory colony maintained in the Entomology Laboratory of the Instituto de Investigaciones Agropecuarias y Forestales (IIAF), Universidad Michoacana de San Nicolás de Hidalgo (UMSNH), Mexico. Larvae were individually reared in 30 mL plastic cups and fed on a formaldehyde-free artificial diet [25]. Adults were transferred to brown paper bags (18 × 11 × 40 cm) for mating and provided with a 15% honey solution as food. After the onset of oviposition, paper bags were replaced daily to ensure proper egg collection. The colony was maintained in a climatic chamber (Lumistell^MR^, Celaya, Mexico) at 25 ± 2 °C, 65 ± 5% relative humidity (RH), and a photoperiod of 16 h:8 h (L:D).

### 2.2. Fungal Isolate

An isolate of *B. bassiana* (Bb-IIAF1-24) was obtained from a naturally infected larva of the ghost moth, *Phassus huebneri* (Geyer) (Lepidoptera: Hepialidae), collected from a blackberry (*Rubus* sp.) crop located in Los Reyes, Michoacán, Mexico (19°34′59.17″ N, 102°30′40.89″ W). This isolate was cultured on potato dextrose agar (PDA; Bioxon^®^, Mexico City, Mexico) and incubated at 25 °C and 75% HR, in the dark for 7 days. This isolate was identified as *B. bassiana* based on the morphological characteristics, using the keys developed by Humber [26]. For preservation, the isolate was stored in 10% glycerol (JT Baker^®^, Radnor, PA, USA) at 4 °C in the entomopathogenic fungi collection of the Insect Pathology laboratory, IIAF, UMSNH, El Trébol, Tarímbaro, Michoacán, Mexico.

### 2.3. Fungal Culture

To propagate the Bb-IIAF1-24 isolate, a polysporic culture was initially established on PDA. Conidial viability was subsequently assessed through a germination test. For this, microcultures were prepared using 14-day-old conidia obtained from the polysporic culture [27]. Germination was evaluated after 24 h of incubation, and the percentage of germinated conidia was calculated according to Rombach [28]. Conidia were considered germinated when the germ tube length reached at least twice the length of the conidium.

After conidial viability determination, monosporic cultures were obtained from the original isolate based on the procedure described by Goettel and Inglis [29], with the modification that incubation was conducted at 25 °C instead of 28 °C. Conidia from a monosporic culture of 20 days old were harvested by scraping them into 100 mL of 0.05% Tween^®^ 80 (Sigma Aldrich^®^, Darmastadt, Germany) and homogenized for 1 min using a vortex mixer. Conidial suspensions were quantified using a Neubauer hemocytometer (Hausser Scientific^®^, Horsham, PA, USA) and diluted to a final concentration of 1 × 10^8^ conidia mL^−1^ for the bioassays.

### 2.4. DNA Extraction

Genomic DNA was extracted from fresh mycelium of Bb-IIAF1-24 isolate. To obtain sufficient biomass, three 5 mm agar plugs from a 17-day-old culture grown on PDA were individually transferred into 100 mL of Sabouraud Dextrose Broth (SDB; Bioxon^®^, Mexico City, Mexico). Liquid cultures were incubated at 25 °C in darkness for 3 days under agitation at 120 rpm using an orbital shaker (Thermo Fisher Scientific^®^, Waltham, MA, USA).

Mycelial biomass was subsequently recovered by filtration [30], rinsed twice with sterile water, and stored at −80 °C until further processing. For DNA extraction, approximately 20 mg of mycelium from each liquid cultures were individually placed into 2 mL microcentrifuge tubes and mixed with preheated CTAB extraction buffer (Sigma Aldrich^®^, Darmstadt, Germany) (65 °C). These samples were homogenized using a micropestle (Eppendorf^®^, Hamburg, Germany), incubated at room temperature for 5 min and DNA was extracted using phenol–chloroform–isoamyl alcohol (Sigma Aldrich^®^, Darmstadt, Germany) (25:24:1). DNA was then precipitated by the addition of 100 µL of 10 M sodium acetate and 500 µL of isopropanol and stored at −20 °C for 24 h. The DNA sample was pelleted by centrifugation at 14,000 rpm for 5 min at 8 °C and the pellet was washed with 70% ethanol, air-dried and finally resuspended in 50 µL of sterile water. DNA concentration and purity were determined using a NanoDrop Lite spectrophotometer (Thermo Fisher Scientific Inc., Waltham, MA, USA) [27].

### 2.5. PCR Amplification and Sequencing

Amplification of the *β-tubulin* gene was performed by polymerase chain reaction (PCR) using the primers TUB-F (5′-TGG GCY AAR GGY CAC TAC ACY GA-3′) and TUB-R (5′-TCA GTG AAC TCC ATC TCR TCC AT-3′). The β-tubulin gene is commonly used to elucidate taxonomic relationships in entomopathogenic fungi [31,32]. The primers were designed to amplify a fragment of approximately 838 bp. Each reaction was carried out in a total volume of 50 µL containing 25 µL of GoTaq^®^ Green Master Mix (Promega, Madison, WI, USA), 1.25 µL of each primer, and ~10 ng of genomic DNA.

Cycling conditions consisted of an initial denaturation of 2 min at 95 °C followed by 10 s at 95 °C, 30 s at 50 °C, 1 min at 72 °C, 34 cycles for 10 s at 95 °C, and a final extension for 5 min at 72 °C. PCR products were observed by electrophoresis in a 1% agarose gel prepared with SB buffer (Sigma Aldrich^®^, Darmstadt, Germany) (1 M boric acid, 0.25 M sodium hydroxide, pH 8.5). Amplification products were stained with ethidium bromide (Thermo Fisher Scientific, Waltham, MA, USA) and visualized using a GelDoc EQ imaging system (Bio-Rad Laboratories, Hercules, CA, USA) with Image Lab software v5.2.1 (Bio-Rad Laboratories). Fragment size was estimated with reference to a 100 bp DNA ladder (Jena Bioscience, Jena, Germany).

Amplified products were purified using the Wizard SV Gel and PCR Clean-Up System (Promega^®^, Madison, WI, USA) and subjected to bidirectional Sanger sequencing at the Laboratorio Nacional de Genómica para la Biodiversidad (LANGEBIO, Irapuato, Mexico) using the same primer pair. Forward and reverse sequences were assembled and manually edited to generate a consensus sequence using Geneious v2013.1.2 (Biomatters, Auckland, New Zealand). The consensus sequence was compared using the Basic Local Alignment Search Tool (BLAST^®^) against the National Center for Biotechnology Information nucleotide (NCBI) data base (https://www.ncbi.nlm.nih.gov). Sequences with the highest similarity were downloaded in FASTA format to be used as references in the phylogenetic analyses.

The resulting sequence was deposited in the GenBank database of the NCBI under accession number PX935148 (www.ncbi.nlm.nih.gov/nuccore/PX935148).

The *β-tubulin* gene sequence from the Bb-IIAF1-24 isolate was compared with four *B. bassiana* sequences retrieved from GenBank, including sequences from Colombia (AY366063), Japan (AB830334), the United Kingdom (AJ312228), and the United States of America (USA) (DQ079603). The sequences of *Cordyceps javanica* from Taiwan (MN215882), *Cordyceps fumosorosea* from Mexico (KT225603), *Isaria farinosa* from USA (DQ079607), and *Colletotrichum siamense* from South Korea (PP704653) were used as outgroups to root the tree. All sequences were aligned in MEGA 12 using the MUSCLE algorithm. A phylogenetic tree was inferred using the Neighbor-Joining (NJ) method implemented in MEGA 12, employing Tamura–Nei + Gamma distribution model (TN93+G) substitution model and uniform rates among sites. Branch support was assessed using 1000 bootstrap replicates.

Based on the final alignment, pairwise genetic distances were estimated using the TN93+G substitution model. The resulting distance matrix comprised corrected pairwise distances among all sequences included in the study and was analyzed in R (version 4.5.2) for heatmap construction using the pheatmap package v1.0.13.

### 2.6. Experimental Procedure

Untreated seeds of a maize cultivar obtained locally were pre-germinated in trays lined with paper towels moistened with sterile distilled water. Two pre-germinated seeds with a visible coleoptile were sown in humus-rich soil and sand (3:1, *v*/*v*) in an upright polyethylene bag (15 cm wide × 30 cm high). To minimize variations in plant development, one seedling per pot was eliminated five days after sowing. Plants were grown under greenhouse conditions. Irrigation was carried out three times per week with 150 mL of water adjusted to neutral pH using a laboratory pH meter (Ohaus^®^, Nänikon, Switzerland). The irrigation volume (150 mL per plant) was selected to maintain adequate soil moisture throughout the experiment without causing waterlogging. Each plant was soil fertilized once a week with three grams of a granular NPK fertilizer (17-17-17) (Hydro Environmet^®^, Tlalnepantla, Mexico) per week. Although the physicochemical properties of the soil were not determined in this study, the use of a substrate composed of humus-rich soil mixed with sand supported adequate maize plant growth, consistent with previous reports [33].

Four weeks after sowing, groups of nine maize plants (=1 replicate) were subjected to one of the following treatments: (1) foliar application of the Bb-IIAF1-24 isolate, (2) soil application of the Bb-IIAF1-24 isolate, (3) foliar application of water (control), and (4) soil application of water (control). The Bb-IIAF1-24 isolate was applied at a concentration of 1 × 10^8^ conidia mL^−1^ in sterile distilled water containing 0.05% Tween 80. Control plants were treated only with sterile distilled water containing 0.05% Tween 80. Treatments were randomly assigned, and there were three replicate groups per treatment. Previously, five second-instar *S. frugiperda* larvae (12 h after molting) were placed into the whorl of each maize plant using a fine brush. Fungal applications were performed at 6:00 PM.

For foliar application, 5 mL of the conidial suspension was sprayed onto each maize plant using a hand-held sprayer. During foliar application, the soil surface in each polyethylene bag was covered with aluminum foil to prevent runoff of the conidial suspension on to the soil. For soil application of the Bb-IIAF1-24 isolate, 5 mL of conidial suspension was sprayed directly onto the soil around the base of each maize plant, covering an approximately 10 cm circular area surrounding the stem.

After foliar and soil application, maize plants were individually placed in a muslin cloth sleeve (200 μm mesh) to confine the *S. frugiperda* larvae. Plant height was measured from the soil surface to the whorl, and stem width was measured at the soil level. Measurements were recorded daily over an 8-day period. Subsequently, the larvae were recovered using sterile forceps and the feeding damage was assessed using the defoliation scale described by Kaya et al. [34], where grade 1 corresponded to 0–10% defoliation, grade 2 to 11–25%, grade 3 to 26–75%, and grade 4 to 76–100% defoliation.

### 2.7. Assessment of Endophytic Colonization

All maize plants from each treatment described in Section 2.6 were sampled to evaluate endophytic colonization by the Bb-IIAF1-24 isolate. To this end, leaves, stem, and root of each plant were separated and then gently washed to remove attached soil. Subsequently, these organs were surface-sterilized by immersion in 1.5% sodium hypochlorite (JT Baker^®^, Radnor, PA, USA) for 3 min, followed by 70% ethanol (Helsam S.A. de S.V., Morelia, Mexico) for 2 min, and then rinsed three times with 100 mL of sterile distilled water. To verify the effectiveness of the sterilization procedure, 100 µL volume of the final rinse water was plated onto PDA, according to Parsa et al. [35].

Plant tissues showing visible damage after the sterilization process were excluded. For each plant, one leaf from the whorl, an 18 cm basal stem section, and the entire primary root system were collected.

Six tissue segments (~1 × 0.5 cm for leaf and stem; 1 cm long for root) were excised from each organ. This procedure was performed in triplicate for each organ. Tissue segments were placed onto PDA supplemented with 0.05% streptomycin and chloramphenicol (250 mg L^−1^, *w*/*v*) (Sigma Aldrich^®^, Darmstadt, Germany) to reduce bacterial contamination. Plates were incubated at 25 °C and 75% RH under complete darkness for 14 days. Tissue segments showing mycelial growth were considered positive for fungal colonization. After sporulation, fungal isolates were morphologically identified as *Beauveria* sp. using the keys of Humber [26]. The percentage of endophytic colonization was calculated as the proportion of tissue fragments exhibiting fungal growth, according to Tossou et al. [22]:

Colonization (%) = (number of colonized segments/total number of segments) × 100.

### 2.8. Statistical Analysis

Data were tested for normality and homoscedasticity using the Shapiro–Wilk and Levene’s tests, respectively, prior to analysis. Differences in the endophytic colonization of the Bb-IIAF1-24 isolate between foliar and soil application were analyzed using a generalized linear model (PROC GLM) with a multifactorial design. The factors ‘isolate application’ and ‘organ’ were included. Means were separated using the least squares means test (LSMEANS; *p* < 0.05). Data of plant growth (plant height and stem diameter) and leaf damage were analyzed using a one-way analysis of variance (ANOVA), followed by the Tukey’s HSD test (*p* < 0.05) for mean separation. All statistical analyses were performed using SAS/STAT software (version 8.1; SAS Institute, Cary, NC, USA). Phylogenetic analyses were conducted separately as described in Section 2.5 using the Neighbor-Joining method with the TN93+G substitution model and 1000 bootstrap replicates.

## 3. Results

### 3.1. Molecular Characterization of Beauveria bassiana Isolate Bb-IIAF1-24

**Figure 1:**
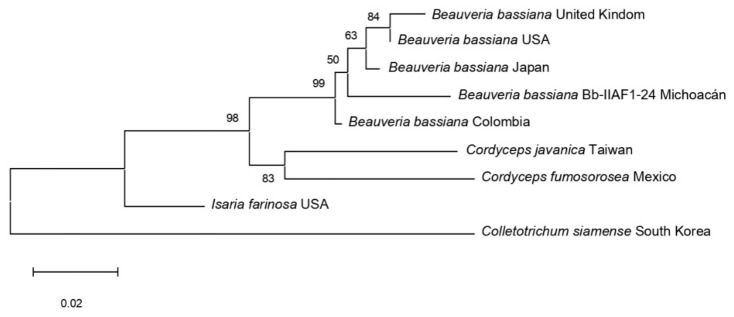
Phylogenetic tree inferred using the Neighbor-Joining method based on nine *β-tubulin* sequences from entomopathogenic fungi. The sequences were clustered applying the Tamura–Nei model with Gamma distribution (TN93+G). Branch support values are based on 1000 bootstrap replicates. The scale bar indicates the number of nucleotide substitutions per site.

Phylogenetic analysis revealed two well-supported clades with bootstrap support values ranging from 83 to 99% (Figure 1). The upper clade included all *B. bassiana* isolates, in which the Bb-IIAF1-24 isolate showed a moderate relationship (50% bootstrap support) with isolates from Japan, the USA, and the United Kingdom. The Colombian isolate formed a separate subclade. Another clade comprised sequences of *C. javanica* and *C. fumosorosea*, whereas the *I. farinosa* sequence appeared as a distinct external lineage.

**Figure 2:**
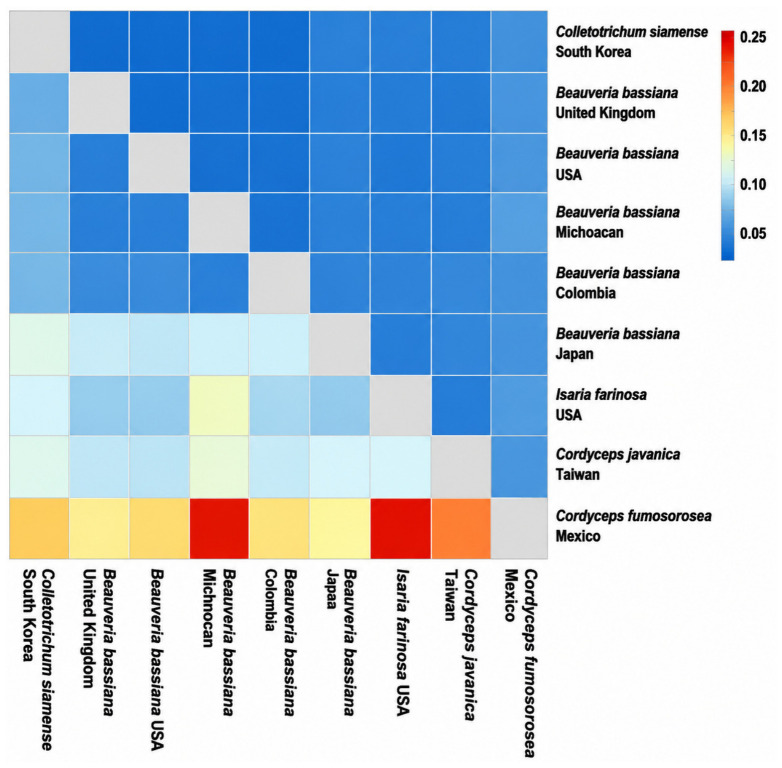
Heatmap of genetic distances among *β-tubulin* sequences of entomopathogenic fungi including the *B. bassiana* Bb-IIAF1-24 isolate from Micoacán, Mexico.

The heatmap based on the genetic distance matrix showed pairwise distances among the analyzed isolates (Figure 2). The *B. bassiana* isolates exhibited low genetic distances among themselves, including the Bb-IIAF1-24 isolate from Michoacán and those from Colombia, Japan, the USA, and the United Kingdom. In contrast, *I. farinosa*, *C. javanica*, *C. fumosorosea*, and *C. siamense* showed higher genetic distance values relative to the *B. bassiana* isolates.

### 3.2. Endophytic Colonization

**Figure 3:**
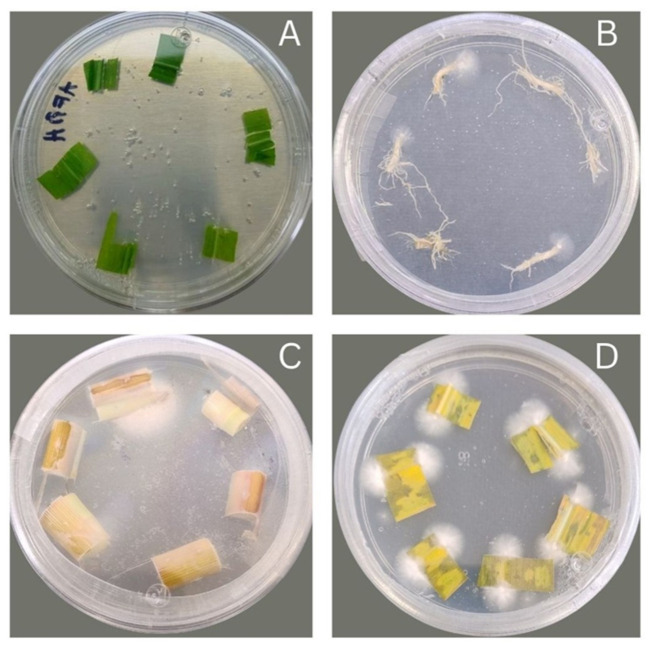
Colonization ability of *B. bassiana* Bb-IIAF1-24 isolate as an endophyte in maize plants. Maize leaves placed on PDA following the surface sterilization procedure (**A**); external growth of the fungus from root (**B**), stem (**C**), and leaf (**D**) sections.

**Figure 4:**
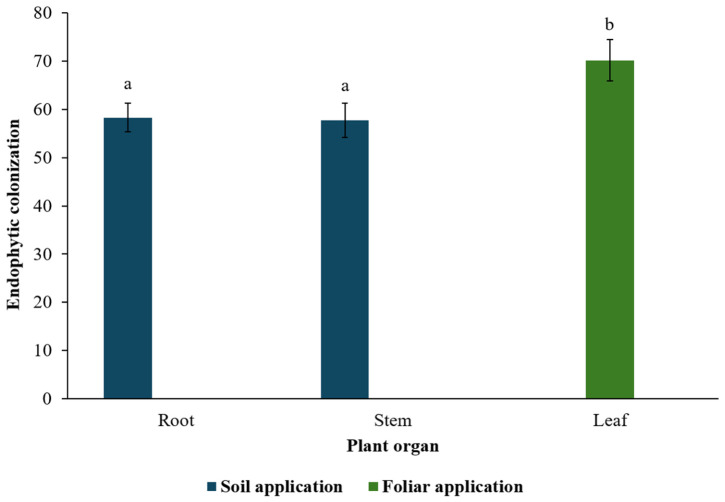
Endophytic colonization (% ± SE) by *B. bassiana* Bb-IIAF1-24 isolate in maize roots, stems and leaves. Different letters above bars indicate significant differences among treatments according to LSMEANS test (*p* ≤ 0.05).

Endophytic colonization of Bb-IIAF1-24 isolate was detected in roots, stems, and leaves of maize plants (Figure 3), and the percentage of colonization varied significantly among these organs (F_2,6_ = 3.67, *p* = 0.026). Isolate application also affects the endophytic colonization (F_3,6_ = 322.5, *p* < 0.0001); foliar spraying resulted exclusively in leaf colonization, with 70.14% ± 4.29 of leaf segments colonized, whereas no colonization was detected in roots or stems (Figure 4). In contrast, soil application resulted in colonization of roots (58.29 ± 2.97%) and stems (57.74 ± 3.58%), but not of leaves. No fungal growth was observed in the sterilization control, and no endophytic colonization was detected in the control plants, indicating that colonization was associated exclusively with fungal inoculation treatments. Endophytic colonization frequency was calculated as the proportion of tissue fragments exhibiting fungal growth.

### 3.3. Effect of the Inoculation Method on Maize Plant Growth and S. frugiperda Herbivory

**Figure 5:**
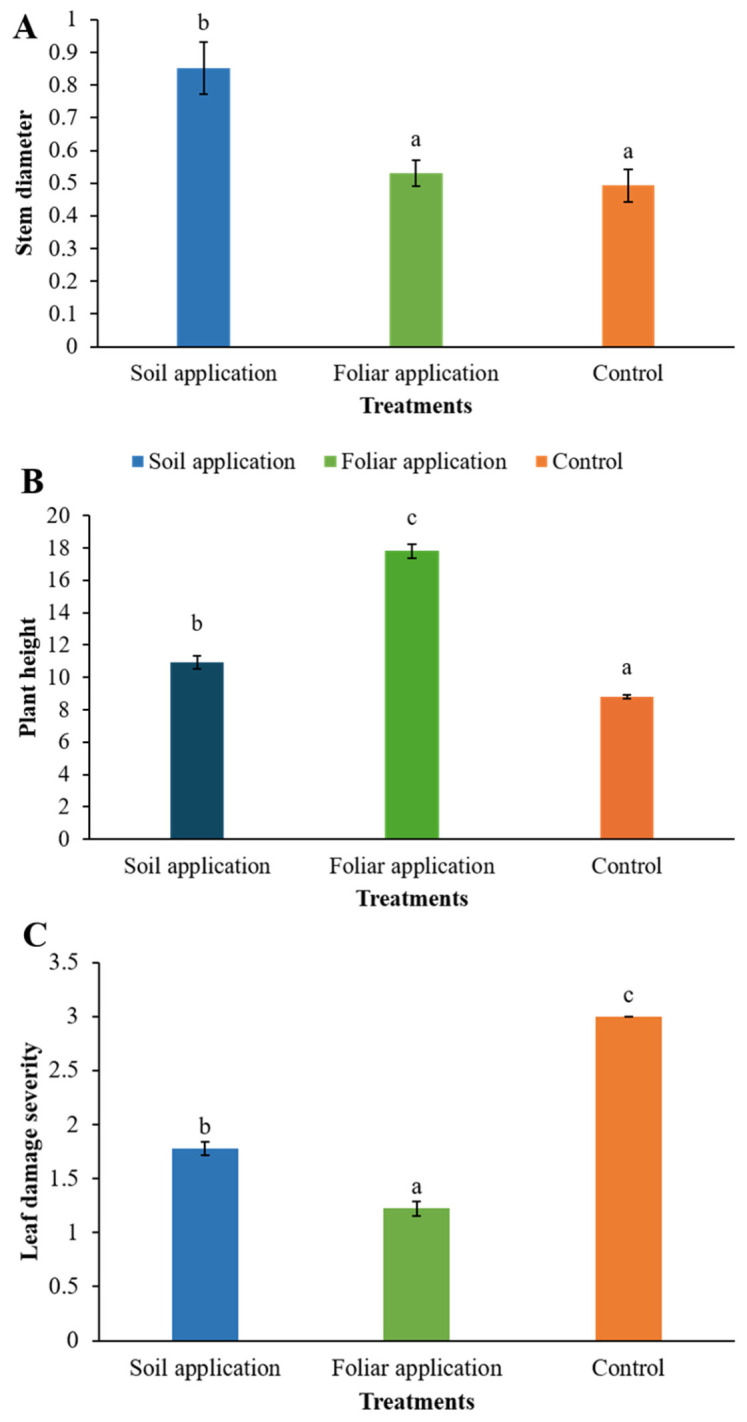
Effects of soil and foliar application of *B. bassiana* Bb-IIAF 1-24 isolate on maize plant growth and *S. frugiperda* herbivory (cm ± SE). Stem diameter (**A**), plant height (**B**) and leaf damage (Scale 0–5) (**C**). Different letters above bars indicate significant differences among treatments based on Tukey’s HSD test (*p* < 0.05).

Plants subjected to soil application developed significantly thicker stems than those treated with foliar sprays or the control (Figure 5A). No significant differences were detected between foliar spraying and the controls (F_2,6_ = 23.52; *p* < 0.0001). In contrast, plants subjected to foliar spraying were significantly taller than those receiving a soil application or the control plants (F_2,6_ = 188.78; *p* < 0.0001; Figure 5B). Both soil (1.78 ± 0.06) and foliar (1.22 ± 0.06) applications exhibited significantly lower foliar damage than the control treatment (3 ± 0.0) (Figure 5C). Moreover, foliar spraying resulted in significantly lower leaf damage than soil spraying (F_2,6_ = 301.5, *p* = 0.0001).

## 4. Discussion

### 4.1. Molecular Characterization of Beauveria bassiana Isolate Bb-IIAF1-24

The phylogenetic analysis and genetic distance heatmap revealed a close genetic relationship among *B. bassiana* isolates, including the Michoacán isolate Bb-IIAF1-24. Nevertheless, differences in branch lengths and pairwise genetic distances indicate genetic heterogeneity among isolates, a pattern widely reported in this fungal species and frequently associated with geographic origin, host range, and ecological adaptation [36,37,38,39]. Similar findings were reported in studies involving 11 native *B. bassiana* isolates from Colombia, Thailand, and the Philippines, where ITS, *β-tubulin* sequences, and amplified fragment length polymorphism (AFLP) markers revealed low but significant intraspecific genetic diversity among strains [40]. Moreover, ISSR and *β-tubulin* analyses of the fungal entomopathogen *Metarhizium rileyi* (Metchnikoff) Sorokin showed considerable genetic heterogeneity among isolates from different geographic regions and hosts [31]. Such variation likely reflects population structuring rather than taxonomic divergence [41,42]. Since our genetic analysis focused on a single marker, a multilocus phylogenetic analysis is still required to confirm the taxonomic relationships between our native isolate and other *B. bassiana* isolates. The clustering of all *B. bassiana* isolates within a single clade was consistent with their taxonomic identity, whereas the separation of *Cordyceps* spp. and *Isaria farinosa* reflected their phylogenetic divergence from *B. bassiana*.

Genetic variability among *B. bassiana* isolates may also have practical implications because differences among strains have been associated with variation in virulence, environmental tolerance, endophytic competence, and adaptation to specific hosts or ecological conditions [43,44,45,46]. Therefore, accurate molecular characterization of native isolates is an important step for selecting strains with potential for biological control and plant protection programs.

### 4.2. Endophytic Colonization

Endophytic colonization of *B. bassiana* Bb-IIAF1-24 isolate was detected in all evaluated maize plant organs, confirming the ability of this fungus to establish within maize tissues. However, the leaves showed the highest colonization. This pattern suggests that aerial tissues may provide more favorable conditions for fungal persistence, potentially due to differences in microenvironment, nutrient availability, or tissue structure [15,41,42,47,48,49,50,51]. In contrast, foliar application of two *B. bassiana* isolates (BS195 and BNE20) in cucumber plants resulted in lower endophytic establishment compared to the root soaking method [52]. The lack of significant differences between roots and stems, as well as among treatments within each organ, indicates that the fungus can systemically colonize the plant regardless of the application method, although its distribution within the plant is not uniform [47,48,51].

The ability of *B. bassiana* to colonize multiple plant organs is particularly relevant for its potential use in biological control programs, as systemic colonization may increase the persistence of the fungus within the host plant and extend its protective effects against herbivorous insects and plant pathogens [53,54,55,56]. Furthermore, successful establishment in aerial tissues may enhance interactions between the fungus and above-ground feeding insects [57,58], potentially contributing to the reduction in herbivory observed in the present study.

### 4.3. Effect of the Inoculation Method on Maize Plant Growth and S. frugiperda Herbivory

Previous studies have reported that entomopathogenic fungi can influence plant growth and repel herbivorous insects, thereby offering an effective strategy for pest management in agricultural crops [13,19,59]. In our study, the observed effects on plant growth parameters indicated that the responses vary significantly depending on the *B. bassiana* application method, which agrees with other studies [16,60,61,62]. Soil application resulted in increased stem diameter, which may be associated with localized interactions in the rhizosphere that enhance nutrient uptake or modify plant physiology [63,64]. In contrast, foliar application promoted greater plant height than soil application, suggesting that above-ground inoculation may more directly influence shoot development [16,51,64,65]. Similarly, several authors have demonstrated the effects of different *B. bassiana* strains on plant growth promotion in crops such as *Z. mays* [66], *Triticum aestivum* L. [63,67], *Oryza sativa* L. [51], *Phaseolus vulgaris* L. [68], *Vicia faba* L. [65], and *Solanum lycopersicum* L. [69]. The contrasting responses observed in our study highlight that different application methods can differentially influence specific growth traits. These differences may be related to the distinct routes through which the fungus interacts with plant tissues [58,70,71]. Soil inoculation primarily favors root-associated colonization and rhizosphere interactions, whereas foliar inoculation may facilitate more direct establishment in aerial tissues. Consequently, the physiological responses induced by *B. bassiana* may differ according to the site of colonization, resulting in variable effects on plant growth parameters [22,72,73]. Foliar application may be the most effective inoculation method for enhancing plant growth, although its effects may not be consistent across all developmental parameters. The combined use of these methods could promote both above-ground and root growth, although further studies are needed to confirm this.

In this study, both application methods reduced the leaf feeding damage caused by *S. frugiperda* larvae compared to the control, indicating a protective effect associated with the presence of the *B. bassiana* Bb-IIAF1-24 isolate. The lower damage observed under foliar application is consistent with its high effectiveness in reducing herbivory, which may be linked to greater fungal activity in aerial tissues where feeding occurs [74,75]. Soil application also reduced damage, although to a lesser extent, suggesting that root-associated colonization can still contribute to plant defense, possibly through indirect mechanisms [47]. Several studies have suggested that endophytic entomopathogenic fungi may reduce herbivory through multiple mechanisms, including the production of insecticidal or deterrent metabolites within plant tissues, induction of plant defense responses, and alterations in plant nutritional quality that negatively affect insect feeding behavior and performance [72,76,77]. These mechanisms may act individually or synergistically depending on the fungal isolate, host plant, and insect species involved [70,78]. Sui et al. [14] also showed that maize plants inoculated with *B. bassiana* were significantly less damaged by *Ostrinia furnacalis* (Guenée) (Lepidoptera: Crambidae) larvae compared to non-inoculated plants. In addition, these authors determined that inoculation of *B. bassiana* improvement significantly the plant biomass and yield. Similar effects have been observed when maize plants were inoculated with *M. anisopliae* [59]. The greater reduction in leaf damage observed following foliar inoculation may also be related to the higher levels of fungal colonization detected in leaves, which could increase the likelihood of direct interactions between the fungus, plant tissues, and feeding larvae. These findings suggest that, beyond its role as an entomopathogen, *B. bassiana* may contribute to plant growth promotion and improved crop performance through physiological and nutritional benefits.

## 5. Conclusions

Molecular characterization and phylogenetic analysis confirmed the taxonomic identity of the *B. bassiana* isolate Bb-IIAF1-2024. Pairwise genetic distance analysis revealed that this isolate exhibited low genetic variability compared to other isolates from different geographic locations. The Bb-IIAF1-2024 isolate successfully established as an endophyte in maize plants and influenced both plant growth and herbivory, with these effects depending on the inoculation method. These findings highlight the potential of native *B. bassiana* isolates as endophytic agents for promoting plant growth and managing insect pests. However, our study was based on a single molecular marker and conducted under greenhouse conditions. Therefore, additional multilocus analyses and field studies are needed to confirm the genetic relationships with other isolates and to evaluate practical applications of this fungus in maize production systems.

## Data Availability

The sequence was submitted to the GenBank public collection of the National Center for Biotechnology Information (NCBI). The accession number is shown in Section 2.5. The raw data supporting the conclusions of this article will be made available by the authors on reasonable request.

## Abbreviations

The following abbreviations are used in this manuscript:

ANOVA	Analysis of Variance
RH	Relative humidity
L:D	Light:Dark
PDA	Potato Dextrose Agar
